# Bacterial Cell Division Regulation by Ser/Thr Kinases: A Structural Perspective

**DOI:** 10.2174/138920312804871201

**Published:** 2012-12

**Authors:** Alessia Ruggiero, Paola De Simone, Giovanni Smaldone, Flavia Squeglia, Rita Berisio

**Affiliations:** 1 Institute of Biostructure and Bioimaging, CNR, Via Mezzocannone, 16. I-80134, Napoli, Italy; 2 Department of Chemistry, University of Naples “Federico II”, I-80134 – Via Cinthia 4, Napoli, Italy

**Keywords:** Cell division, structure, phosphorylation, peptidoglycan.

## Abstract

Recent genetic, biochemical and structural studies have established that eukaryotic-like Ser/Thr protein-kinases are critical mediators of developmental changes and host pathogen interactions in bacteria. Although with lower abundance compared to their homologues from eukaryotes, Ser/Thr protein-kinases are widespread in gram-positive bacteria. These data underline a key role of reversible Ser/Thr phosphorylation in bacterial physiology and virulence. Numerous studies have revealed how phosphorylation/dephosphorylation of Ser/Thr protein-kinases governs cell division and cell wall biosynthesis and that Ser/Thr protein kinases are responsible for distinct phenotypes, dependent on different environmental signals. In this review we discuss the current understandings of Ser/Thr protein-kinases functional processes based on structural data.

## 1 INTRODUCTION

Reversible protein phosphorylation is a critical instrument to transfer signals from environments and regulate cellular functions, such as cell division and cell wall biosynthesis. The involvement of eukaryotic-like serine/threonine kinase (STPKs) during cell division and cell wall biosynthesis is well documented [1,2]. STPK and their associated phosphatases (STPP) play major regulatory roles in eukaryotes [3] and in prokaryotes [4,5], particularly in Gram-positive bacteria. Extensive studies have shown that STPKs play essential roles in cell competence, biofilm production [6], cell shape/division [7], cell envelope biosynthesis [7,8], sporulation [9,10] and stress response [11]. The first reported characterization of a bacterial STPK was made in the soil microorganism *Myxococcus xanthus* [12,13], but similar kinases have been reported in *Streptococcus agalactiae* [14], *S. pneumoniae* [15-17], *S. pyogenes* [18,19], *S. mutans* [6], and *Bacillus subtilis* [9], as well as *Mycobacterium tuberculosis *[20].

STPKs belong to the protein kinase family named as one-component signal transduction systems. Unlike the two-component systems, which are composed of two dedicated proteins (a sensor and a regulator), one-component systems combine both sensing and regulating properties [21]. Usually, these properties reside in two distinct domains, sensory and regulatory, with different cellular localization. Recent comparative genomics analyses suggest that the majority of prokaryotic signal transduction systems consist of one-component systems and lack phosphotransfer domains, typical of two-component systems. Furthermore, signaling through STPKs appears to be the dominant prokaryotic signaling system [21].

Acting both as sensors and regulators, STPKs typically share a modular structural organization, in which the sensing domain is extracellular and is connected to an intracellular kinase domain by a transmembrane linker [22]. Sequence analyses and recent structural data show that the extra-cellular regions of many STPKs contain more copies of small domains, denoted as PASTA (Penicillin binding protein And Ser/Thr kinase Associated) domains. As its name suggests, these domains were previously found in penicillin-binding proteins, where they were suggested to be involved in cell wall biosynthesis [23].

Mycobacteria and other Actinomycetes encode several eukaryotic-like kinases (namely PknA-L). Recently, PknB from *M. tuberculosis* (Mtb) has become one of the most studied STPKs [24-28]. Also, structures of inactivated mutants and the PknB kinase domain in a complex with an ATP-competitive inhibitor have suggested key insights into the regulation mechanism of this class of enzymes [27]. Due to the high homology of the PknB kinase domain with kinases from other Gram-positive bacteria, PknB constitutes a useful model to understand the enzymatic properties of STPKs. A lower level of structural information is available for the sensor extra-cellular domains of STPKs and most of the available structural information was achieved only recently [29-32]. This review will focus on recent structural findings of STPKs, specially focusing on those from human pathogens. Several excellent reviews are available for a more general discussion of STPKs [33,34].

## 2 MOLECULAR PLAYERS IN BACTERIAL CELL DIVISION

During the cell cycle and in preparation for division, bacteria replicate their DNA and segregate the newly formed chromosomes. A division septum then assembles at a predetermined site between the chromosomes, the cell constricts and ultimately, the mother cell splits into two identical daughters due to septum degradation [35-37]. A major constituent of bacterial septa and of the whole cell wall is peptidoglycan (PGN), an essential cell wall polymer, formed by glycan chains of β-(1-4)-linked-N-acetylglucosamine (GlcNAc) and N-acetylmuramic acid (MurNAc) cross-linked by short peptide stems. Depending on the amino acid located at the third position of the peptide stem, PGN is classified as either Lys-type or meso-diaminopimelic acid (DAP)-type. 

Bacterial cell growth and cell wall biosynthesis are mediated by a collection of proteins whose action is tightly coordinated at the level of septal ring [38]. In *E. coli*, cell division takes place at the mid-cell after the chromosomal replication and segregation into two daughter nucleoids. After the completion of chromosome segregation, the division process begins with the formation of the septal ring, called Z-ring, a polymer of the tubulin-like protein FtsZ [39]. FtsZ is almost universally conserved and has also been identified in Mtb as one of the major cytoskeletal organizers of the mycobacterial divisome [40,41]. The depletion of FtsZ from bacteria results in long filamentous cells [42]. The ring formed by FtsZ involves the highly ordered recruitment of both structural and enzymatic proteins involved in peptidoglycan synthesis and thus in the formation of the septum [43]. In the most studied rod-shaped bacteria, such as *E. coli *and* B. subtilis*, inhibitory mechanisms mediated by either the Min system [44-46] and the nucleoid occlusion system [36,47,48] have been proven to prevent the assembly of the Z ring on top of unreplicated chromosomal DNA. 

**Fig. (1):**
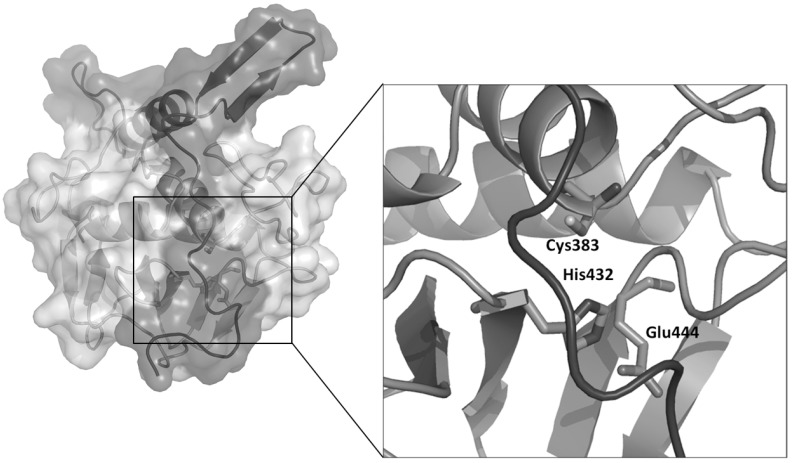
Cartoon and surface representation of the crystal structure of RipA from Mtb [54]. The catalytic and regulatory domains are reported in light and dark grey, respectively. The inset shows an enlargement of the catalytic site residues, locked by the regulatory domain.

Septal PGN is initially shared between daughter cells and must be degraded by PGN hydrolases to complete the division process. Whereas as many as 18 hydrolases are known to be involved in septum cleavage of *E. coli*, only few hydrolases are known in mycobacteria, which possess a unique envelope structure with additional layers of arabinogalactan and mycolic acids [40,49]. Cell separation is mediated in Mtb by the essential NlpC/P60 endopeptidase RipA (Resuscitation promoting factor Interacting Protein), which cleaves peptidoglycan peptide crosslinks [50], similar to other cell separating endopeptidases, like CwlT from *B. subtilis* [51] and Spr from *E. coli *[52]. RipA has a remarkable effect on the bacterial phenotype, since *ripA* depletion strains in *M. smegmatis* exhibit a decreasing growth and an abnormal phenotype, consisting in branching and chaining bacteria [53]. Crystallographic studies of RipA have yielded new insights in the functional regulation of this enzyme (Fig. **1**). Indeed, the crystal structure clearly reveals a zymogenic nature of RipA, a finding which is confirmed by cell wall degradation assays [54]. Interestingly, RipA co-localizes at bacterial septa with the resuscitation promoting factor RpfB [55], a key cell wall hydrolase involved in Mtb resuscitation from a state of low metabolism denoted as dormancy [56-58]. Furthermore, it has been shown that the PGN hydrolase activities of the two enzymes synergize, although the structural basis of this synergistic action is hitherto not clear [59]. This synergy can be inhibited by the interaction of RipA with the penicillin-binding protein PBP1, a key PGN synthase [60]. It is therefore tempting to believe that interactions between RipA, RpfB and PBP1 allow Mtb to coordinate the processes of PGN synthesis and PGN hydrolase activity during cell division. 

## 3 STPKs IN BACTERIAL CELL DIVISION

Cell division and cell wall synthesis are closely linked complex phenomena and play a crucial role in the maintenance and regulation of bacterial growth and virulence [61]. Excellent work has examined the physiological role of protein kinases in cell division and growth in human pathogens [1,62,63]. For example, mycobacterial STPKs *pknA* and *pknB* are organized in an operon that encodes other essential proteins involved in cell shape (Wag31) and envelope biosynthesis (RodA, PbpA) [64] and whose transcription is noticeably high during exponential growth [65]. Furthermore, overexpression or depletion of *pknB* or *pknA* genes alters cell phenotypes in different mycobacterial strains. In particular, mycobacterial cells in which *pknB* o *pknA* gene transcription was partially inhibited are highly elongated. These morphological changes have provided the evidence that these two kinases are key regulator of active cell replication and cell shape in mycobacteria [65]. 

The role of PknA in regulating cell division in mycobacteria was also confirmed in another study showing modulation of FstZ activity by PknA [66]. Furthermore, also cell wall synthetic enzymes such as MurD, GlmU, and PbpA appear to be regulated by PknA or PknB, supporting the strong relation existing between STPK-dependent phosphorylation and peptidoglycan biosynthesis in cell elongation [67-69]. 

The availability of complete genome sequences has confirmed the presence of genes encoding PknB-like proteins in a broad range of gram-positive bacteria, whose genes number differs greatly from that observed in mycobacteria and other Actinomycetes. For example, *S. pneumoniae *and* B. subtilis* possess only one and two STPKs, respectively. Despite these differences, bacterial STPKs sequences are more similar to each other than to their human homologues, with which they share low sequence identity (<30%) [70]. Recent progress has been made to elucidate the biological function of StkP in *S. pneumoniae* and *B. subtilis* cell division. The kinase StkP from *S. pneumoniae* is essential for bacteria survival and virulence [15] and, similar to PknB from Mtb [71], it localizes at the cell division sites and its localization depends on extracellular PASTA domains [17]. In *B. subtilis*, the kinase PrkC is responsible for resuscitation from dormancy induced by the presence of PGN fragments in the bacterial *milieu *[72]. The wide distribution of PknB-like proteins in the genomes of gram-positive bacteria suggests that STPKs regulatory function in cell shape and division is widely preserved among prokaryotes.

## 4 STRUCTURAL ORGANIZATION OF STPKs: A STATISTICAL SURVEY

**Fig. (2):**
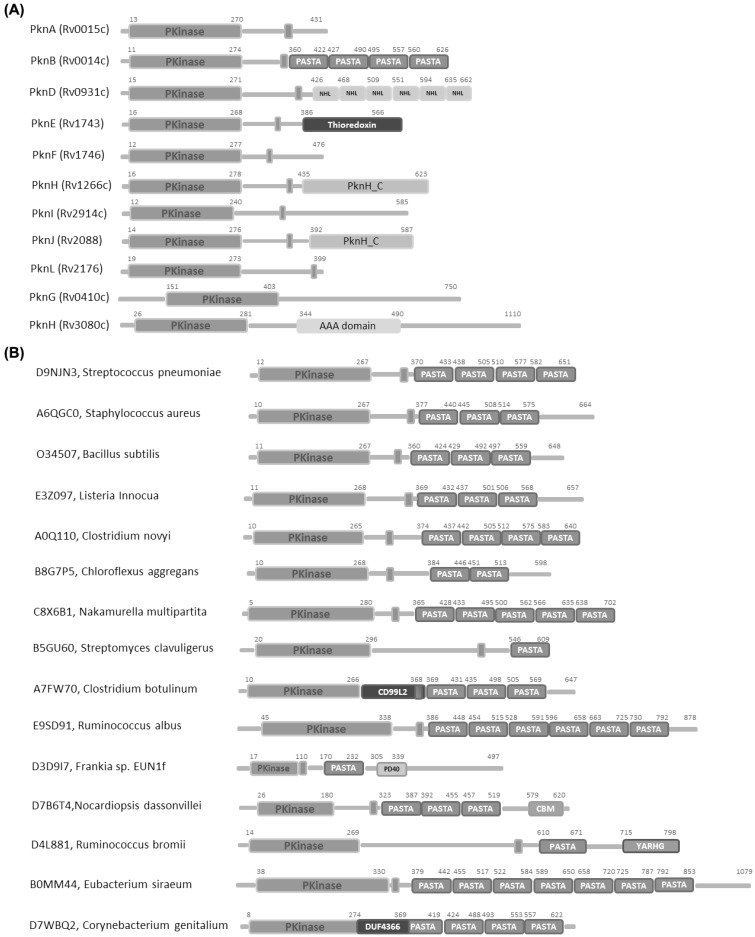
Domain organizations of (A) STPKs from Mtb and (B) PASTA domain containing STPKs. Each box refers to a different protein domain, as defined by the PFAM database [106].

Bacterial STPKs reveal high diversity in their domain organizations. A significant number of them contain trans-membrane spanning segments, different than eukaryotic Ser/Thr kinases (Fig. **2**). The frequent occurrence of trans-membrane kinases (TM-kinases) in most bacterial species is in line with their twofold role as receptor like kinases involved in direct interaction with the extra-cellular ligands [21]. TM kinases encoded in diverse groups of Gram-positive bacteria often contain extra-cellular PASTA domains (Fig. **2B**), whose strong conservation in STPKs suggests a key role in conserved signaling pathways. Crystallographic studies of STPKs in the last decade have helped understanding key mechanisms of regulation and substrate recognition (Table **1**). Twenty-six structures of portions of STPKs have been currently deposited with the Protein Data Bank, most of them of the intra-cellular kinase domains (Table **1**).

## 5 STRUCTURAL FEATURES OF BACTERIAL STPK KINASE DOMAIN

**Fig. (3):**
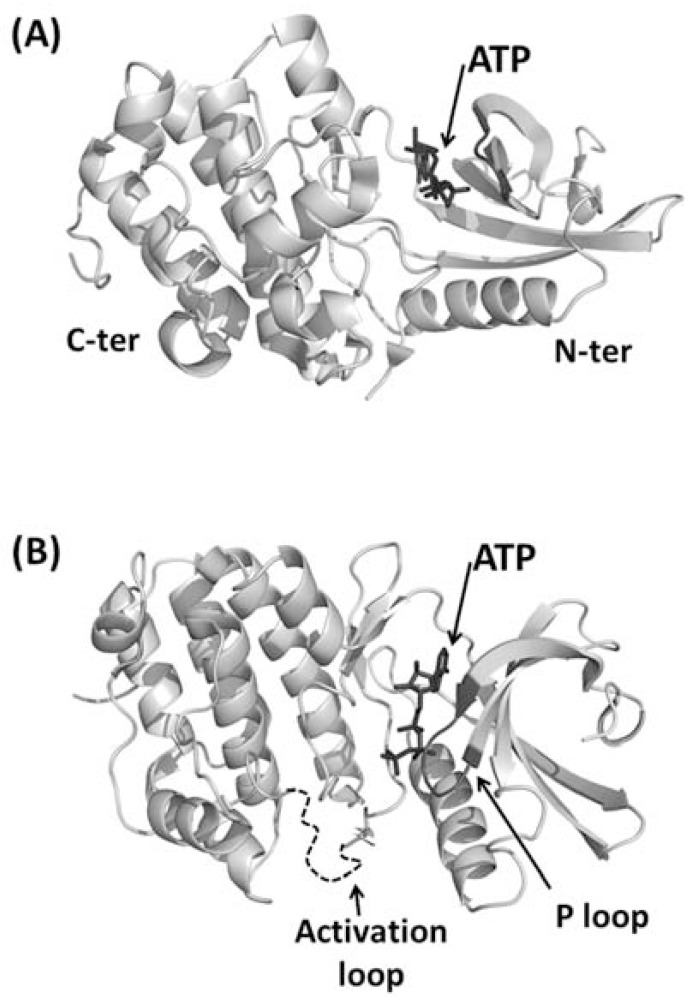
Cartoon representation of the kinase domain from PknB of M. tuberculosis. (A) Side view. N-terminal and C-terminal lobes are indicated with N-ter and C-ter, respectively. ATP is represented in ball-and-stick and indicated by an arrow. (B) Top view. The P loop is represented in black whereas the activation loop, which is disordered in the crystal structures, is represented as a dashed curve.

Two pioneering crystallographic studies of bacterial STPK kinase domain (KD) described complexes of PknB KD with two different ATP analogues [24,28]. These studies showed that the overall fold consists of two lobes: an N-terminal subdomain, including a curled β-sheet and a long α-helix, and a C-terminal lobe composed by α-helices (Fig. **3**). On analogy with eukaryotic kinases, these structures were defined as exhibiting a “closed” conformation, due to the relative orientation between both the N- and C-terminal lobes. A critical role in the control of the activity of protein kinases as well as in the recognition of their substrates is played by the activation loop (residues 164-177 in PknB). Furthermore, the hexapeptide 18-GFGGMS-23 forms a loop (P-loop, Fig. **3**) in which the main chain amides of the glycines coordinate the phosphates.

These structures have for the first time shown the conformation similarity of bacterial STPKs with eukaryotic Ser/Thr kinases. Indeed, conserved structural features comprise the enclosure of the N- and C- terminal lobes around the nucleotide, the P loop and the catalytic loop in which residues involved in phosphate transfer are located (Fig. **3**). These similarities support the conservation of mechanisms of nucleotide binding and catalysis in both bacterial and eukaryotic STPKs [24,28].

One main feature makes bacterial STPKs different from their eukaryotic cognates: the conformation of the activation loop. In eukaryotes, the activation loop of the inactive unphosphorylated state, denoted as ‘off’ state, either blocks the active site or is not structured. Differently, in the phosphorylated state, the ‘on’ state, the activation loop adopts a specific conformation that allows ATP to enter the active site and takes part itself to the catalytic binding cleft. In bacterial STPKs, the activation loop remains disordered even in the phosphorylated state of both Holo and Apo forms, suggesting that it might be a general feature of mycobacyerial protein kinase [34,76]. Despite this feature suggests inactivity of this conformational state of the kinase domain, *in vitro* studies have demonstrated that, like in eukaryotes, phosphorylation activates the enzyme [24]. A clear solution to this puzzle has hitherto not been formulated. Possibly, STPK KD domains need a still unknown additional substrate or cofactor to achieve full stabilization of the activation loop [28]. However, more structural information is still awaited for a full understanding of structural basis of STPK activation.

Unlike most eukaryotic STPKs, the KD of PknB forms stable homodimers, characterized by a conserved interface present in the N-terminal lobe. Different independent crystal forms of PknB exhibit this feature, suggesting that Mtb receptor kinases are activated by reversible interactions through its N- lobe interface [24-26,28]. In agreement with this notion, the structure of the KD of apo-PknE has shown the presence of a similar dimerization interface [76]. Remarkably, structurally related dimers allosterically activate some human STPKs, like PKR, a cytosolic dsRNA-dependent antiviral protein kinase [78] and Ire1, the bifunctional transmembrane kinase/endoribonuclease, which is involved in the unfolded protein response [79]. The first direct biochemical demonstration that N-lobe dependent dimerization activates autophosphorylation and transphosphorylation through an allosteric mechanism in STPKs was established for PknD of Mtb [80]. Different N-lobe mutants were structurally characterized to check whether PknB dimerization through its N-lobe increases the activity of the enzyme [26]. These studies showed that the loss of dimer interface in these PknB variants destabilizes the active site, which adopts an inactive conformation. Therefore, N-lobe dimerization in bacterial STPK stabilizes the active KD conformation, confirming an allosteric mechanism of activation [26]. Recent studies have also provided new understanding on regulatory mechanisms in cell division mediated by STPKs, as they have shown that the STPK kinase PknB from Mtb is able to phosphorylate a kinase-like domain in the essential peptidoglycan biosynthetic protein MviN by recruiting a fork head-associated domain protein, FhaA. The crystal structure of MviN in complex with the FHA domain suggests that FHA mediates the formation of a regulatory complex with PknB [77].

Given to the broad range of crucial cellular processes in which STPKs are involved, these proteins represent strong candidates for the development of novel drugs. Indeed, partial depletion of *pknA* or *pknB* in Mtb results in narrow and elongated cells [65]. The low similarity between bacterial and human STPKs (<30%) can be directly exploited for drug design, as it can increase the inhibitor selectivity. Recently, a high throughput screening approach was used to identify small molecules targeting PknB kinase. A total of 54 000 compounds (diverse compound and kinase-focused collection) were tested measuring the *in vitro* phosphorylation of GarA (Rv1827) by PknB [81,82]. This study has permitted the identification of a new class of ATP competitive inhibitors with IC50s in the nM range [27]. However, improved inhibitors are still awaited, since their MICs values against Mtb growth within macrophages were found to be only in the micromolar range, likely due to limited cell wall permeability [27].

## 6 STRUCTURAL FEATURES OF STPK EXTRA-CELLULAR REGION

Despite the proven key role of STPKs in regulating environmental responses in bacteria and therefore the importance of the extracellular portions of these proteins (sensor domains) in sensing environmental changes, a molecular explanation for this process is still under debate. Also, only limited structural information on sensor domains is available. 

**Fig. (4):**
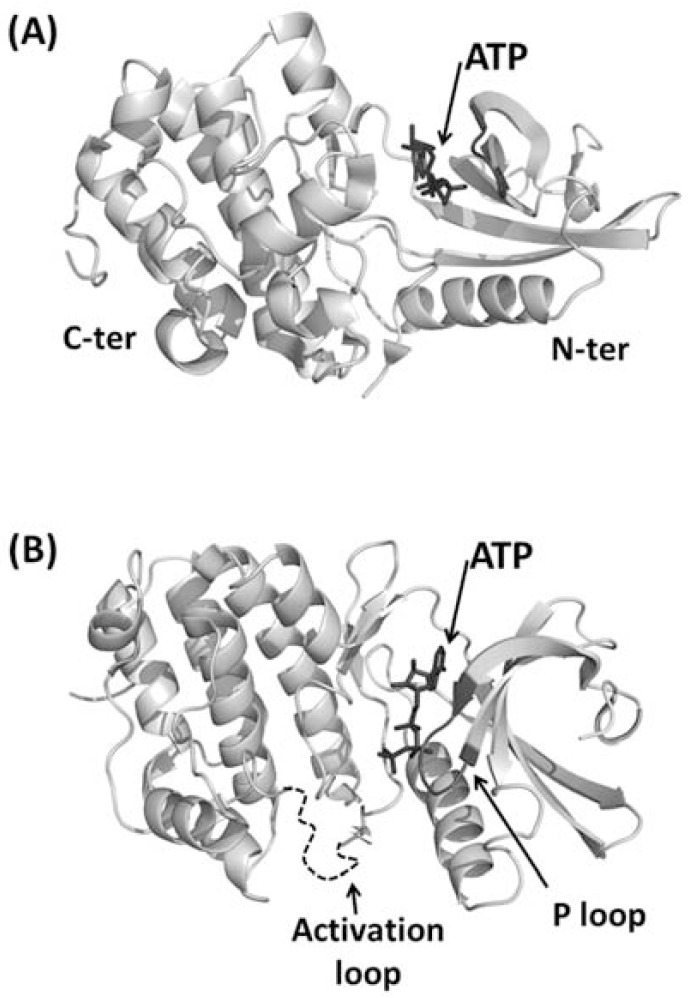
Cartoon representations of available sensor domains. In particular, panels A-D report structures of sensor domains of PknD from Mtb, PknB from Mtb, PrkC from *S. aureus* and PknH from Mtb, respectively.

The extracellular domain of the STPK PknD from Mtb was shown to form a highly symmetric six-bladed β-propeller (Fig. **4A**). The authors suggested that regulatory ligands could bind the central part of the PknD β-propeller, denoted as the “cup”, and that binding could change the protein quaternary structure [31]. However, no clues were given about the nature of the regulatory ligands [31]. The same authors determined the crystal structure of PknH sensor domain, which adopts an unanticipated fold containing two intramolecular disulfide bonds and a large hydrophobic and polar cleft. The conservation of residues lining the cleft and those surrounding the disulfide bonds has suggested that PknH binds a small-molecule ligand. Also in this case, however, the nature of these regulatory molecules and the mechanism underlying kinase activation is unknown [32].

Parallel studies have led to the structure determination of two STPK PASTA-domain containing extracellular regions, PknB and PrkC from the human pathogens Mtb and of *S. aureus*, respectively [30,71]. PknB consists of an intracellular catalytic kinase domain, a transmembrane domain and an extracellular region, which has four PASTA domains (Fig. **2**). An overall linear shape of PknB extracellular region was determined by combining the NMR structural description of three overlapping portions PknB (corresponding to PASTA domains 1-2, 2-3, and 3-4) with small-angle X-ray scattering (SAXS) experiments of the whole extracellular PknB (Table **1** and Fig. **4B**). 

PrkC from *S. aureus* is predicted to have a Ser/Thr kinase domain (residues 1-270), a region of unknown structure and function (residues 271-377) that includes a transmembrane helix (residues 349-373), and an extra-cellular region (residues 378-664, Fig. **2B**). The crystal structure of the extracellular portion of PrkC revealed that it consists of four consecutive domains, arranged sequentially such that only neighbouring domains interact with each other (Fig. **4C**). Consistent with PknB solution structural studies [71], the structure of extracellular PrkC shows that the three PASTA domains display a linear and regular organisation. In this organisation, each domain exhibits twofold symmetry with respect to its neighbouring domains (Fig. **4C**) [30]. Interestingly, beside the three predicted PASTA domains, the structure reveals the existence of a fourth domain, at the C-terminal end of the molecule, not predicted by searches in the PFAM database (Fig. **2B**)[83]. Although sequence analyses against the Protein Data Bank do not identify any significant homolog for this domain, a DALI search [84] revealed a structural similarity to Immunoglobulin (Ig)-like domains (s-type Ig-fold, Fig. **4C**) [85]. A comparative analysis of the structure of this domain with other Ig-fold domains clearly unveils that the Ig-domain of PrkC lacks the N-terminal strand. Although further studies are needed to define the role of this new type of incomplete Ig-fold domains, it has been suggested that the exposure of an anomalously large number of backbone β-strand hydrogen-bond donors and acceptors may endow these domains with adhesive properties [86-88]. On analogy with the *E. coli* pilus subunit PapG, PrkC Ig-like domain may be involved in peptidoglycan binding [30,85]. Consistently, sequence alignments indicate that this incomplete Ig-fold domain is present in other proteins involved in bacterial sporulation [30,85].

## 7 STPK PASTA-DOMAIN CONTAINING EXTRACELLULAR REGIONS ARE MUROPEPTIDE SENSING ANTENNAS

With the discovery that PrkC, a PASTA domain-containing STPK kinase from *B. subtilis*, is essential for resuscitation from dormancy induced by muropeptides [72], it has become clear that the extracellular PASTA domains serve as sensors for peptidoglycan fragments. Notably, *B. subtilis* spores germinate in response to DAP-type (diaminopimelic acid) muropeptides, which constitute *B. subtilis* cell wall, but not in response to L-Lys type muropeptides. This finding suggested that extra-cellular domains of PrkC exhibit specificity of muropeptide binding. However, the ability of muropeptides to physically bind the extracellular region of the protein was only very recently assessed [71,89]. 

In the last year, two studies have investigated the quantitative binding of muropeptide fragments to the extracytoplasmic regions of PknB from Mtb [71] and PrkC from *B. subtilis* [89]. These studies have demonstrated that PGN fragments bind the extracytoplasmatic region of these two kinases, and have defined molecular requirements for ligand binding. Indeed, the critical role of DAP in binding has been evidenced in both studies, consistent with the DAP-type structure of the stem peptide present in both mycobacterial and *B. subtilis* cell walls [71,89]. Mir *et al.* further showed that PknB is preferentially localized to the septum than to the cell poles, the sites of active PGN synthesis in mycobacteria, and that the PASTA domains of PknB are required for its localization [71]. Consistent with these findings, STD NMR spectroscopy clearly revealed that strongest binding involves the DAP residue [89]. The key involvement of the DAP residue in protein recognition well agrees with the previous finding that only muropeptides containing DAP in their peptide stem resuscitate *B. subtilis*, whereas L-Lys-type muropeptides do not [72]. A further achievement of these studies was the identification of the muropeptide binding site on PrkC [89]. Indeed, they showed that recognition occurs through interactions of DAP with the Arg500, since a mutation of this aminoacid in the PrkC completely impaired muropeptide binding [89]. This finding agrees well with the key role played by arginine in the specific recognition of DAP-muropeptides by Peptidoglycan Recognition Proteins [90]. In this scenario, the key role of Arg500 in binding provides a clear explanation for the ability of PrkC from *B. subtilis *to discriminate between DAP- and Lys-type muropeptides in bacterial revival [72]. Using this mechanism, *B. subtilis* bacteria, which possesses a DAP-type PGN, can cross-talk and trigger resuscitation by its own cell wall turnover [89]. 

## 8 THE PASTA DOMAIN: A MUROPEPTIDE-BINDING DOMAIN?

PASTA domains exist in penicillin-binding proteins [23]. The first structural characterization of this domain has been reported for the penicillin binding domain PBP2x from *Streptococcus pneumoniae*, which contains two C-terminal PASTA domains, each of them consisting of an alpha helix and three beta strands[91]. A further structural characterization of PBP2x has been made in presence of cefuroxime, α β–lactam antibiotic mimicking the unlinked peptidoglycan [92]. In this structure (PDB 1QMF), cefuxomine binds one PASTA domain, a finding which has suggested that PASTA domains might bind unlinked peptidoglycan [23]. The structural studies recently emerged prove that PASTA domains do have the ability to bind muropeptides [71,89]. However, binding studies on the sensor domain of PrkC have shown that only one of the three PASTA domains is endowed with muropeptide-binding properties [89]. This finding proves that muropeptide binding ability of PASTA domains is not an intrinsic property of these domains but it strongly depends upon the local composition of the putative muropeptide-binding site.

## 9 CURRENT UNDERSTANDING OF STPK ACTIVATION THROUGH STRUCTURAL STUDIES

The kinase domain of STPKs is highly homologous among several bacterial species. Common to PknB [93], PrkC from *B. subtili*s [94] and PrkC from *S. aureus *[95], the kinase domain undergoes self-phosphorylation. As previously discussed, the x-ray structure of PknB kinase domain suggests a model in which a structural and functionally asymmetric “front-to-front” association occurs. This dimerization mode leads to the phosphorylation of serine and threonine residues located in the kinase activation loop (Fig. **3**) [25,96]. Therefore, sensor domains of STPKs must transmit their signals, e.g. muropeptide binding, by helping dimerization of the intracellular kinase domain. 

**Fig. (5):**
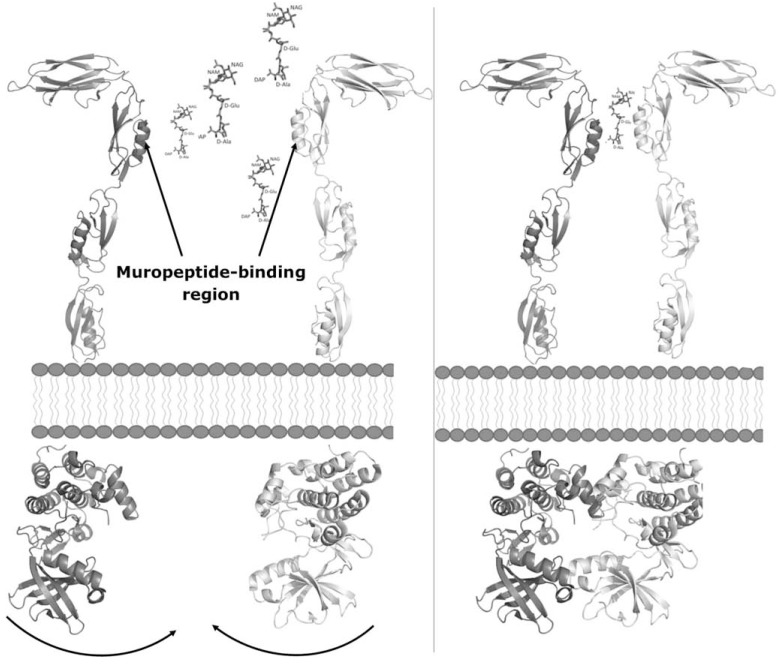
A *naive* model STPK activation mediated by muropeptides. Left: muropeptides bind to PASTA3 on the PrkC sensor domain [89]. Right: muropeptide binding bridge protein-protein interaction and brings the intracellular kinase domains close enough to allow for their dimerization. The model of the entire STPK is based on the crystal structures of PrkC sensor from *S. aureus* (PDB code 3PY9) and of PknB kinase domain (PDB code 3F69). The muropeptide interaction site on the PASTA3 domain of the PrkC sensor region is based on Squeglia et. al [89].

Four structural descriptions of STPK sensor domains are hitherto available. The sensor-domain structures of PknD and PknH of Mtb adopt globular structures [31,32] whereas the structures of PASTA-containing sensor domains adopt completely different structures [29,30]. PknB and PrkC sensor domains share elongated and multi-domain structures (Fig. **4**) containing either three (PrkC) of four (PknB) PASTA domains [29,30]. These structural arrangements contrast with previous modeling studies [24], based on the crystal structure of PBP2x from *S. pneumoniae*, which suggested an antiparallel arrangement of the PASTA domains. On the other hand, the observed linear organization of PASTA domains is fully compatible with a muropeptide-dependent dimerization mechanism underlying STPK activation (Fig. **5**) [72,89]. However, although muropeptide binding to both the sensor domains of PrkC from *B. subtilis* [89] and PknB from Mtb [71] were proven and the interaction site was mapped for PrkC [89], both sensor domains were found to be unable to form dimers *in vitro* [29,30]. This observation points to a more complex protein dimerization mechanism, which may involve STPK trans-membrane portions. A suggestive hypothesis to investigate further was proposed by Mir *et al.* [71] stating that the muropeptide binding ability of kinases is important for their localization at the septum and the cell poles, where local concentrations of muropeptides are high. The recruitment of high concentrations of kinases at these sites results in high concentrations of the intracellular kinase domain and therefore kinase activation through dimerization [71].

## CONCLUDING REMARKS

Commonly used drugs target structural features and metabolic characteristics of prokaryotes that are significantly different from those in eukaryotic cells. Drugs used to treat bacterial diseases can be grouped into categories based on their modes of action. In general, these drugs inhibit cell wall synthesis [97,98], protein synthesis [99-101], or nucleic acid synthesis [102,103]. However, the emergence of multidrug-resistant and extensively drug-resistant bacterial infections has made the development of new and effective therapies an urgent need. In this scenario, processes governing host-pathogen interactions are a strong opportunity to develop molecular entities of therapeutic interest.

There is increasing evidence that STPKs are key mediators of developmental changes and host-pathogen interactions in bacteria [11,104]. While progress has been made in understanding the involvement of STPKs at different cellular levels [7-11], new information is awaited to understand their molecular mechanisms of activation. Indeed, it is well established that their extra-cellular sensor domains regulate the catalytic activity of STPKs [13][14]. However, the mechanisms involved in the regulation by the sensor domains as well as the signals that are sensed by the different kinases are not fully clarified. 

The recent structural information on STPKs has provided insights into the involvement of STPK kinases in cell division processes. Indeed, the modular and linear organization of PASTA-domain containing STPK sensors and their ability to bind muropeptides [29,30,71,89] have corroborated the hypothesis that cell wall hydrolysis and cell division modulation by STPKs are tightly connected [72]. Although the interaction site remains unknown for PknB, it has been shown that binding of PknB to muropeptides is responsible for its localization to the bacterial septum and poles [71], where the concentration of muropeptides is high due to the action of cell wall hydrolases [54,105]. It is however, under debate whether muropeptide binding induces dimerization of extra-cellular portions of STPKs or if activating dimerization of intracellular kinase domains is due to a high concentration of STPKs induced by the high concentration of muropeptides at bacterial septa and poles [29,30,71,89]. Together, these data provide a *fil rouge* between cell wall hydrolysis, a process connected with both bacterial growth and resuscitation from dormancy [54,72,105], and STPK regulatory mechanisms *via* post-translational modifications.

## Figures and Tables

**Table 1.** Available STPKs Structures.

Ser/Thr kinase	Source	PDB code	residues/domain	reference
PrkC	S. aureus	3PY9; 3M9G	Extracellular sensor domain (378-664)	[30,73]
PknB	Mtb	3OUV	3th PASTA domain (491-558)	Not published
PknB	Mtb	2KUD ; 2KUE; 2KUF; 2KUI	Extracellular sensor domain (355-626)	[29]
PknD	Mtb	1RWI; 1RWL	Extracellular sensor domain (403-664)	[31]
PknH	*Mtb*	4ESQ	Extracellular sensor domain (435–626)	[32]
PknB	*S. aureus*		Intracellular kinase domain (1-291)	[74]
PknB	*Mtb*	1MRU; 1O6Y; 2FUM; 3ORM; 3ORL; 3ORP; 3ORI; 3ORK; 3ORO; 3ORT; 3F61; 3F69	Intracellular kinase domain (1-308)	[24-28]
PknG	*Mtb*	2PZI	Intracellular kinase domain (74-750)	[75]
PknE	*Mtb*	2H34	Intracellular kinase domain (14-289)	[76]
Rv3910	*Mtb*	3OUK	Intracellular kinase domain (679-963)	[77]
